# A Survey on Secure Computation Based on Homomorphic Encryption in Vehicular Ad Hoc Networks

**DOI:** 10.3390/s20154253

**Published:** 2020-07-30

**Authors:** Xiaoqiang Sun, F. Richard Yu, Peng Zhang, Weixin Xie, Xiang Peng

**Affiliations:** 1 The Guangdong Key Laboratory of Intelligent Information Processing, College of Electronics and Information Engineering, Shenzhen University, Shenzhen 518060, China; xqsun@szu.edu.cn (X.S.); wxxie@szu.edu.cn (W.X.); 2 Key Laboratory of Optoelectronic Devices and Systems of Ministry of Education and Guangdong Province, College of Physics and Optoelectronic Engineering, Shenzhen University, Shenzhen 518060, China; xpeng@szu.edu.cn; 3 Department of Systems and Computer Engineering, Carleton University, Ottawa, ON K1S 5B6, Canada; richardyu@cunet.carleton.ca

**Keywords:** vehicular ad hoc networks, secure computation, homomorphic encryption

## Abstract

In vehicular ad hoc networks (VANETs), the security and privacy of vehicle data are core issues. In order to analyze vehicle data, they need to be computed. Encryption is a common method to guarantee the security of vehicle data in the process of data dissemination and computation. However, encrypted vehicle data cannot be analyzed easily and flexibly. Because homomorphic encryption supports computations of the ciphertext, it can completely solve this problem. In this paper, we provide a comprehensive survey of secure computation based on homomorphic encryption in VANETs. We first describe the related definitions and the current state of homomorphic encryption. Next, we present the framework, communication domains, wireless access technologies and cyber-security issues of VANETs. Then, we describe the state of the art of secure basic operations, data aggregation, data query and other data computation in VANETs. Finally, several challenges and open issues are discussed for future research.

## 1. Introduction

With the help of many different technologies, vehicular ad hoc networks (VANETs) [1,2,3,4,5] are expected to enhance transportation efficiency, reduce accidents, offer great mobility service options and alleviate environmental damage [6,7,8,9]. In the next decade, VANETs will continue to develop steadily and progressively because of improved infrastructure, wireless sensors and communication technologies. The global market of VANETs is one of biggest markets in the world; it is expected to reach $1.5 trillion in 2030. Many countries and large-scale automotive manufacturers are speeding up the design of practical VANETs.

In VANETs, vehicle data are first collected from various sensors. Because these collected data are raw and rough, they cannot be used directly by users. Thus, they must be calculated by a third party with a powerful computation ability. The final computation result can be used in several applications; for example, according to the query requirements, some useful vehicle data may be returned back to the user. Another application is data aggregation, which is helpful to compress, filter and transmit vehicle data.

Vehicle data [10,11,12] include users’ original and real data, such as location, biometric information and so on. The handling of the data usually involves personal privacy, safety of property and even the security of human life. In order to analyze vehicle data, there is a need for a mathematical model to be developed. In addition, vehicle data are disseminated to an untrusted third party for computation; thus, it is likely that vehicle data will be illegally accessed, forged, tampered or discarded in the process of data dissemination [13,14,15] and computation.

In order to protect user privacy, we can encrypt vehicle data by a traditional encryption algorithm. Unfortunately, encrypted vehicle data cannot be analyzed easily and flexibly. Homomorphic encryption [16] makes it possible for encrypted user data to be analyzed by an untrusted Cloud server without decryption. In addition, the communication cost between the user and the Cloud server is low. Homomorphic encryption is helpful for securing precision in medicine, the secure distribution of power in smart grids, the secure prediction of teenagers’ dropout risks and the secure analysis of vehicle data.

Thus, an untrusted third party can analyze encrypted vehicle data perfectly using homomorphic encryption without leaking users’ private data. Finally, the encrypted result is returned to the user. The result of the analysis can be obtained by the user with their own secret key. To analyze vehicle data securely, the key point is to construct a secure and efficient homomorphic encryption scheme. However, the efficiency of homomorphic operations is too low; there is no efficient method for the construction of an efficient secure computation method based on homomorphic encryption. Furthermore, an exploration of several types of secure computation is required.

**Figure 1:**
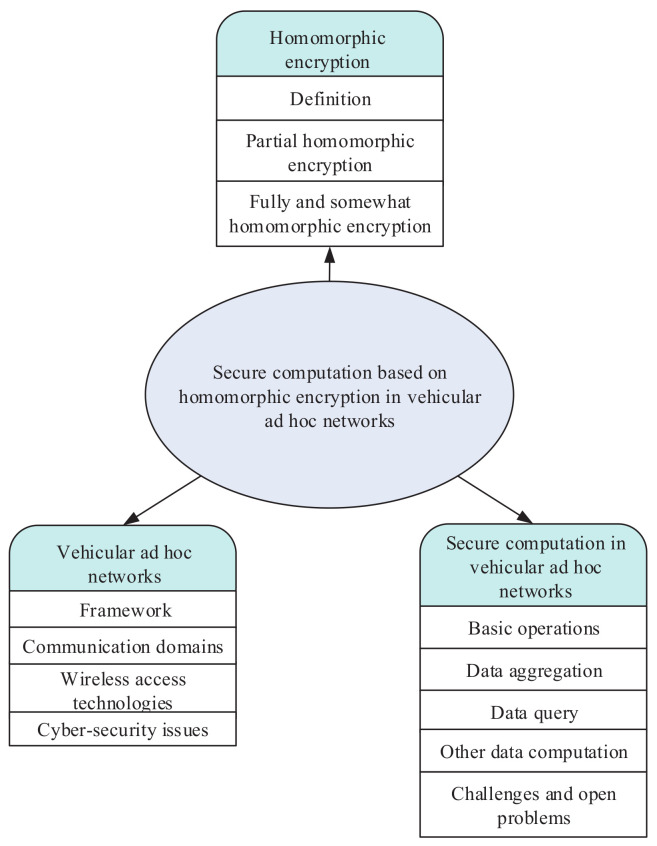
The road map of secure computation based on homomorphic encryption in vehicular ad hoc networks.

In this paper, we provide a brief survey of some of the works that have already been done regarding the construction and analysis of secure computation based on homomorphic encryption in VANETs, as well as the open issues in this field. The taxonomy graph of this paper is presented in Figure 1. As shown in this figure, we identify three aspects of secure computation based on homomorphic encryption in VANETs. These aspects consist of homomorphic encryption, the overview of VANETs and secure computation based on homomorphic encryption in VANETs. At present, there are several surveys [17,18] on traditional homomorphic encryption schemes. To the best of our knowledge, there is no survey on secure computation based on homomorphic encryption in VANETs. We believe that our discussion and exploration can give readers an overall understanding of this important field and will encourage more subsequent studies on the open issues.

The rest of the article is organized as follows. In Section 2, we provide the related definitions and current state of homomorphic encryption. Section 3 introduces the related framework, communication domains, wireless access technologies and cyber-security issues in VANETs. Furthermore, Section 4 presents the state of the art of secure basic operations, data aggregation, data query, other data computation in VANETs and several open issues. Finally, Section 5 concludes the article.

## 2. Homomorphic Encryption

Homomorphic encryption is an encryption technique that supports a particular time-consuming evaluation algorithm. This algorithm allows certain types of operations to be carried out on the ciphertext without requiring access to a secret key. In addition, this algorithm generates an encrypted result in which the decryption matches the result of the computation on the plaintext. For example, there are two plaintexts *x* and *y*; we want to compute 3xy+x without leaking *x* and *y*. Thus, we first use the homomorphic encryption algorithm Enc to encrypt *x* and *y*. Enc(x) and Enc(y) are ciphertexts of *x* and *y*, respectively. Then, we compute Enc(3xy+x)=3×Enc(x)×Enc(y)+Enc(x), where 3×Enc(x)×Enc(y)+Enc(x) denotes homomorphic operations. The final ciphertext is Enc(3xy+x), and the plaintext is 3xy+x. In order to obtain a better understanding of homomorphic encryption, we present related definitions and the current state of homomorphic encryption in this section.

### 2.1. Definition

**Definition** **1**  (Homomorphic Encryption [19]). *A homomorphic encryption scheme HE=(KeyGen,Enc,Dec,Eval) consists of four probabilistic polynomial algorithms. The detailed definition of homomorphic encryption is described as follows:*

- $HE.KeyGen(1^{\lambda })$: The security parameter λ is taken as an input. Output parameters include a public key pk, a secret key sk and an evaluation key evk, namely $\left(pk,sk,evk\right)\leftarrow HE.KeyGen\left(1^{\lambda }\right)$.
- HE.Enc(pk,m): The public key pk and a plaintext m are taken as inputs. Then, the ciphertext c is output, namely c←HE.Enc(pk,m).
- HE.Dec(sk,c): The secret key sk and the ciphertext c are taken as inputs. The decryption result $m^{*}$ is output, namely $m^{*}\leftarrow HE.Dec\left(sk,c\right)$.
- $HE.Eval(evk,f,c_{0},\cdots ,c_{l-1})$: Input parameters include the evaluation key evk, a function f and ciphertexts $c_{0},\cdots ,c_{l-1}$, where the plaintext of $c_{i}$ is $m_{i}$, i=0,⋯,l−1, l is the number of ciphertexts. Then, the final ciphertext $c_{f}$ is output, namely $c_{f}\leftarrow HE.Eval\left(evk,f,c_{0},\cdots ,c_{l-1}\right)$, where $HE.Dec\left(sk,c_{f}\right)=f\left(m_{0},\cdots ,m_{l-1}\right)$, f is an operational circuit over the plaintext space.

**Definition** **2**  (Leveled FHE [20]). *Let HE be a homomorphic encryption scheme. If the depth of the circuit is at most L, then HE is a leveled fully homomorphic encryption (FHE) scheme, where the computational complexity of HE is polynomial, and the upper bound of the ciphertext size of c must be independent of L.*

For a family of homomorphic encryption schemes $\left\{\varepsilon^{d}\right\}$ with different depths, they have the same decryption circuit. In addition, $\varepsilon^{d}$ computes all the circuits compactly. In most cases, the bit-length of the evaluation key evk is the only parameter which depends on the leveled FHE scheme with *L*.

**Definition** **3**  (Semantic Security). *Let HE be a homomorphic encryption scheme. There is a probabilistic polynomial-time adversary, if*

$$\begin{array}{cc} Adv[A] & =Pr[A(pk,evk,HE.Enc(pk,0))=1]-Pr[A(pk,\\ & evk,HE.Enc(pk,1))=1]=negl(k), \end{array}$$

*HE is semantic security, where $\left(pk,sk,evk\right)\leftarrow HE.KeyGen\left(1^{\lambda }\right)$. It can resist a chosen plaintext attack (CPA).*

Homomorphic encryption originates from privacy homomorphism, which was proposed by Rivest et al. [16] for banking applications in 1978. Homomorphic encryption outputs a final ciphertext, the decryption of which is equivalent to the result of the same operations on the plaintext. According to the types and numbers of homomorphic operations, there are three different kinds of homomorphic encryption. As the earliest type of homomorphic encryption, partial homomorphic encryption only supports homomorphic addition or homomorphic multiplication. Somewhat homomorphic encryption supports a finite number of homomorphic addition and homomorphic multiplication operations. Then, fully homomorphic encryption (FHE) supports an infinite number of homomorphic addition and homomorphic multiplication operations. These variants are described as follows.

### 2.2. Partial Homomorphic Encryption

There are several partial homomorphic encryption schemes, which are described as follows. In 1978, Rivest et al. [21] proposed the RSA cryptographic algorithm, which supports homomorphic multiplication. Its security is based on the factorization of a large integer. In 1982, Goldwasser and Micali [22] constructed the GM probabilistic encryption algorithm, which supports homomorphic addition. Its security is based on the assumption of quadratic residue. In 1985, the ElGamal cryptographic algorithm [23], which supports homomorphic multiplication, was constructed based on the problem of the discrete logarithm. In 1999, based on the assumption of decisional composite residuosity, Paillier [24] constructed a cryptographic algorithm that supported homomorphic addition. In 2005, Boneh et al. [25] first proposed a cryptographic algorithm that supported an infinite amount of homomorphic addition and one homomorphic multiplication operation.

### 2.3. Fully and Somewhat Homomorphic Encryption

**Figure 2:**
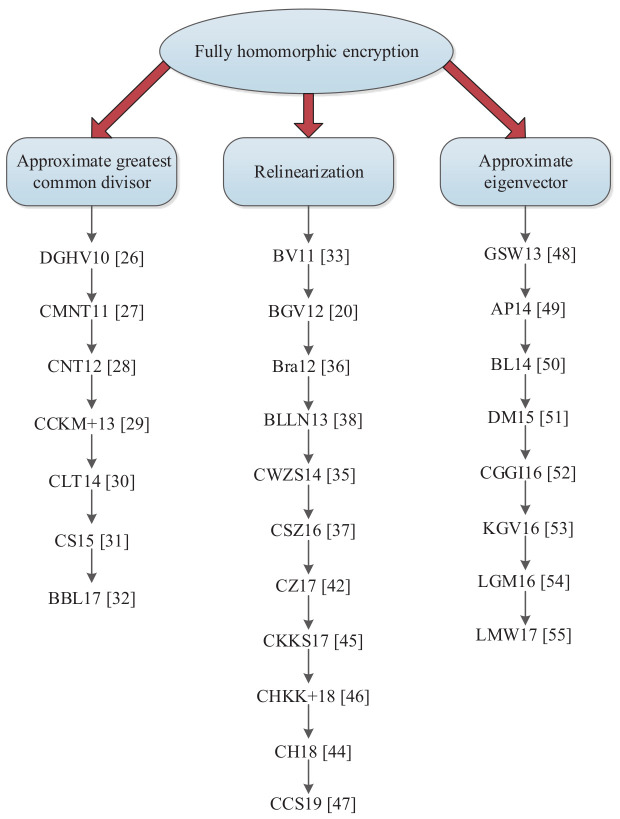
The development of fully homomorphic encryption.

In 2009, based on the theory of ideal lattices, Gentry [19] first constructed a FHE scheme, the security of which was based on problems of bounded distance decoding and sparse subset sums. In Gentry’s scheme, the construction was divided into two parts: the author first proposed a somewhat homomorphic encryption scheme, which could execute low-degree polynomial operations on the ciphertext; then, bootstrapping was realized by the technique of squashing the decryption circuit. The somewhat homomorphic encryption scheme was then converted to a FHE scheme. After that work, FHE became a research hotspot. Numerous experts and scholars around the world have focused on research into FHE. However, the construction of FHE based on the theory of ideal lattices is overly complicated; the sizes of the ciphertext, public keys and private keys are excessively large. Currently, the research into FHE mainly focuses on the techniques of the approximate greatest common divisor (APGCD), relinearization and the approximate eigenvector. The development of FHE is shown in Figure 2, which is described as follows.

#### 2.3.1. FHE Based on APGCD

In 2010, based on the APGCD problem, Dijk et al. [26] first proposed the DGHV10 FHE scheme over integers. Their method only uses trivial operations over integers. In [27], based on the DGHV10 scheme, the proposed somewhat homomorphic encryption scheme, called the CMNT11 scheme, transformed the number of integers from τ to $\sqrt{\tau }$ in the public key. In the CNT12 FHE scheme [28], Coron et al. used the technique of public key compression [27] to reduce the size of the public key. In [20], the technique of squashing the decryption circuit was replaced by the technique of modulus reduction. The computational complexity would increase if the technique of modulus reduction were applied for the DGHV10 scheme. In 2013, Cheon et al. [29] first proposed the batch CCKM+13 FHE scheme over integers by using the Chinese remainder theorem. In 2014, Coron et al. [30] proposed the improved CLT14 FHE scheme over integers, which is scale-invariant, which means that the noise of ciphertexts increases linearly after each homomorphic multiplication. The assumption of learning with errors (LWE) is secure against an attack by a quantum computer. In 2015, Cheon et al. [31] reduced the LWE assumption to the APGCD problem, which means that the hardness of the APGCD problem is not easier than that of the LWE assumption. Based on the improved APGCD problem, Cheon et al. [31] constructed the CS15 FHE scheme without the technique of squashing the decryption circuit. Its ciphertext size is only O(λlogλ), where λ is the security parameter. In 2017, based on the decisional APGCD problem, Benarroch et al. [32] constructed the BBL17 FHE scheme by the technique of approximate eigenvector. The proposed scheme does not require the noise-free component or the assumption of a sparse subset sum.

#### 2.3.2. FHE Based on Relinearization

Based on the LWE assumption, Brakerski et al. [33] constructed the BV11 FHE scheme by the relinearization technique. This scheme abandoned the technique of squashing the decryption circuit and the problem of the sparse subset sum. It could execute one homomorphic multiplication without increasing the ciphertext size. Based on the assumption of binary LWE [34], Chen et al. [35] proposed the CWZS14 FHE scheme, the concrete parameters of which are analyzed as follows. In this scheme, the secret key is generated from ${\left\{0,1\right\}}^{l}$ randomly, where *l* is the dimension. Compared with Brakerski’s FHE scheme [36], in Chen’s scheme, the sizes of the public key and secret key are logq times smaller, the size of tensor ciphertext is log2q times smaller and the key switching matrix is log3q times smaller, where *q* is the modulus. In [37], Chen’s scheme [35] was extended to the batch CSZ16 FHE scheme. Compared with the LWE assumption, the assumption of learning with errors over rings (RLWE) has the advantage that its polynomial multiplication can be implemented by the technique of the fast Fourier transform. Thus, it can be used to construct more efficient FHE schemes, which are described as follows.

In 2012, Brakerski et al. [20] constructed the leveled BGV12 FHE scheme, which uses the technique of modulus reduction to reduce the noise of ciphertext without bootstrapping. In addition, the dimension of ciphertext is reduced by the technique of key switching. The BGV12 scheme supports the technique of single instruction multiple data (SIMD). Based on the variant BGV12 scheme, Helevi [38] built a software library (HElib) that implements FHE by Gentry’s optimization [39]. Brakerski [36] proposed the Bra12 FHE scheme by a new tensoring technique without modulus reduction. This scheme is scale-invariant, which means the same modulus is used in the evaluation process. In 2013, based on [40], Bos et al. [41] constructed the BLLN13 FHE scheme, which supports the SIMD technique. In this scheme, the ciphertext only consists of a single ring element. The ciphertext size is constant after each homomorphic multiplication. Based on the BGV12 scheme, the CZ17 FHE scheme [42] is proposed by using an efficient bootstrapping method, which incurs only polynomial noise $O(n^{3})\cdot B$, where *n* is the dimension of the lattice and *B* is the upper bound of the noise. Based on the FHE scheme in [41], Dowlin et al. [43] implemented a simple homomorphic encryption library (SEAL),which can be used for the secure computation of biological information, genetic data, etc. In the CH18 FHE scheme, Chen et al. [44] improved the homomorphic digit extraction algorithm. Then, the algorithm was applied for the bootstrapping process in the BGV12 scheme. The depth of the bootstrapping process is reduced from logh+2logt to logh+logt, where *h* is the 1-norm ${∥s∥}_{1}$ of the secret key *s* and *t* is the plaintext modulus.

However, the above schemes do not support approximate computation on the ciphertext. Thus, based on the BGV12 scheme, Cheon et al. [45] constructed the CKKS17 homomorphic encryption scheme, which supports approximate homomorphic addition and homomorphic multiplication on the ciphertext. In this scheme, the predetermined precision of message in the ciphertext can be guaranteed by the rescaling procedure. The ciphertext size is decreased significantly. Then, in the CHKK+18 scheme, Cheon et al. [46] proposed an approximate bootstrapping method for the CKKS17 scheme. Next, in the CCS19 scheme, Chen et al. [47] proposed an improved bootstrapping method for the CHKK+18 scheme.

#### 2.3.3. FHE Based on Approximate Eigenvectors

In 2013, Gentry et al. [48] first proposed the simple GSW13 FHE scheme, which was constructed based on the technique of approximate eigenvectors. In the GSW13 scheme, homomorphic addition and homomorphic multiplication are reduced to only addition and multiplication on the matrix. The noise of the ciphertext increases linearly after each homomorphic operation. However, the GSW13 scheme does not support the SIMD technique. Then, based on the GSW13 scheme, some improved FHE schemes were developed, which are described as follows.

In the AP14 FHE scheme [49], Alperin-Sheriff and Peikert improved the GSW13 scheme by symmetric groups and permutation matrices. In this scheme, the speed of bootstrapping is faster than that of the GSW13 scheme. The noise of the ciphertext increases linearly after each homomorphic multiplication. Based on the GSW13 scheme, Berkoff and Liu [50] proposed the leakage-resilient BL14 FHE scheme. The proposed scheme is homomorphic for circuits for which the depth is less than the pre-defined maximum set in the procedure of key generation. In 2015, the DM15 FHE scheme [51] was constructed based on the GSW13 scheme. Ducas and Micciancio used a new method to compute the NAND of two ciphertexts homomorphically. It only takes about 0.5 s to execute the algorithm of bootstrapping on a personal computer. In order to restrict the noise of ciphertext during the procedure of key switching, the DM15 scheme used the binary LWE assumption, where ensuring security proved to be as difficult as the standard LWE assumption. In the CGGI16 FHE scheme [52], Chillotti et al. showed that the internal product can be replaced by a simple external product which consists of ciphertexts which are generated by the GSW13 scheme and the LWE assumption. In this scheme, the time of bootstrapping is reduced to 0.1 s. The size of the bootstrapping public key is decreased from 1 GB to 24 MB under the same security parameter. In the KGV16 FHE scheme [53], Khedr et al. introduced several algorithmic optimizations, which can reduce computational complexity and speed up homomorphic operations for the implementation of the GSW13 scheme. Its parallelism is realized by the GPU platform. The ciphertext size in this scheme is smaller than that of the scheme in HElib. In [54], the proposed LGM16 FHE scheme was made secure against adaptive attacks by a new method. The idea of the method is that a one-time secret key is created each time in the procedure of decryption. Thus, the adversary cannot recover the valid secret key. Based on the GSW13 scheme, Li et al. [55] constructed the multi-bit LMW17 FHE scheme, which could prevent an adversary from obtaining the secret key effectively by a side channel attack.

Unfortunately, in above FHE schemes, ciphertexts are under the same key pair. In order to solve this issue, two kinds of improved FHE schemes—namely multi-key FHE and threshold FHE—have been developed and are described as follows.

Based on the NTRU [56], López-Alt et al. [57] constructed a multi-key FHE scheme. This scheme allows the computation of ciphertexts whose key pairs are different from each other. However, its computational complexity increases exponentially along with the increasing number of key pairs. In 2015, Clear and McGoldrick [58] proposed a multi-key FHE scheme, whose security is based on the LWE assumption in the standard model. Based on the multi-key GSW13 scheme [48], Mukherjeet et al. [59] proposed a multi-key FHE scheme. However, their method only supports homomorphic operations on single-bit plaintext. In this protocol, the ciphertext size grows $n^{2}$ times, where *n* is the number of key pairs. Based on the LWE assumption, Brakerski and Perlman [60] proposed a dynamic multi-key FHE scheme, whose ciphertext size increases linearly along with the increasing number of key pairs. However, the procedure of bootstrapping is needed for the implementation of homomorphic multiplication or homomorphic exclusivity. Based on the LWE assumption, Peikert and Shiehian [61] constructed two kinds of multi-key FHE schemes. The proposed schemes are multi-hop methods for keys, which means that homomorphic operations with other keys can use the former result of homomorphic operations on ciphertexts under a sequence of keys. Besides, the proposed schemes have smaller ciphertext sizes. In the second scheme, ciphertexts are simply GSW13 ciphertexts without any other data. In 2017, based on the RLWE assumption, Chen et al. [62] proposed the first batched multi-hop and multi-key FHE scheme with compact ciphertext expansion. In previous schemes, complicated computations are needed for the expansion algorithm for each ciphertext, whereas Chen’s expansion algorithm only needs to compute evaluation keys. Thus, the complexity of ciphertext expansion only depends on the number of key pairs and has nothing to do with the number of ciphertexts. Based on the GSW13 scheme, Wang et al. [63] proposed a multi-key FHE scheme whose security can be reduced to a some-are-errorless LWE assumption. Based on the LWE assumption, Brakerski et al. [64] proposed a multi-key FHE scheme with a distributed set-up procedure.

Based on the BV11 scheme [33], López-Alt et al. [65] designed a key homomorphic threshold FHE scheme. In this scheme, many key pairs can be combined into a key pair. Based on the GSW13 scheme, Gordon et al. [66] proposed a threshold FHE scheme with flexible and transformed ciphertexts. In this scheme, there is a key pair in the joint key pair; thus, algorithms of encryption and evaluation algorithms need not be changed.

Due to the high efficiency of partial homomorphic encryption and somewhat homomorphic encryption, they have been applied for engineering applications. In FHE, the noise of the ciphertext often increases exponentially after each homomorphic multiplication. Noise reduction is a huge obstacle for the efficiency of FHE. In the FHE schemes over integers, the sizes of the public key and secret key are still excessively large, and homomorphic operations require a great deal of time. Although techniques of modulus reduction and key switching can be avoided in the FHE scheme, which is constructed based on the technique of approximate eigenvector, the speed of homomorphic encryption is effectively improved; however, this scheme only supports homomorphic operation for single-bit plaintext. In the FHE scheme based on the reliearization technique, even the speed of homomorphic encryption is not as fast as that of the FHE scheme based on the technique of approximate eigenvectors, which supports the SIMD technique.

In summary, current research on homomorphic encryption has mainly focused on the design of homomorphic encryption algorithms, the improvement of efficiency for homomorphic encryption and bootstrapping. On the one hand, although homomorphic encryption has developed rapidly in recent years, most homomorphic encryption schemes have been constructed by the technique of relinearization or approximate eigenvectors. New techniques for the construction of FHE have not been created. On the other hand, several experts and scholars have devoted themselves to the study of FHE, but few of them have designed schemes according to the requirements of actual application. Even though the efficiency of FHE is improving, it is still not practical.

## 3. An Overview of Vehicular Ad Hoc Networks

In this section, we describe the related framework, communication domains, wireless access technologies and safety standards in VANETs.

### 3.1. Framework

**Figure 3:**
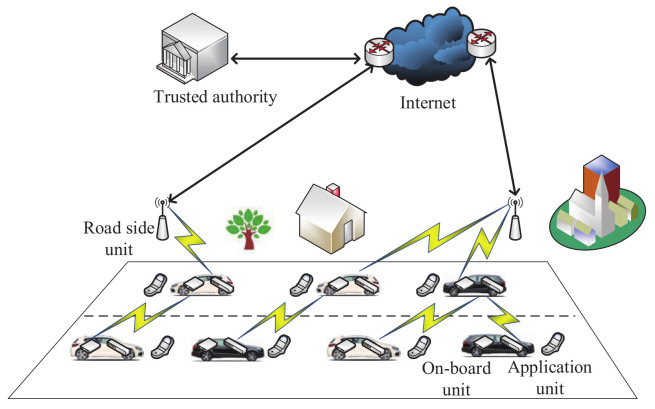
A framework of vehicular ad hoc networks (VANETs).

As shown in a framework of VANETs (Figure 3), the existing entities include the on-board unit, application unit and road-side unit [67]. They are described as follows.

#### 3.1.1. On-Board Unit

Based on the IEEE 802.11p radio protocol [68,69,70], the on-board unit is used to exchange messages with road-side units or other on-board units. It is often equipped in a vehicle. In an on-board unit, there is a resource command processor, a user interface, a special interface which can be used to communicate with other on-board units, a network facility which can be used for short distance wireless communication and resources which include random access memory for storing and retrieving data. The on-board unit can provide services including wireless radio access, ad hoc and geographic routing, the regulation of network congestion, dependable message transmission, data safety and internet protocol mobility.

#### 3.1.2. Application Unit

The application unit is usually embedded in a vehicle. It can be a special device that is used for some secure applications; it can also be a general device—for example, a personal digital assistant. The application unit can communicate with the network only through the road-side unit, which is in charge of all mobility and network functions. The communication between the application unit and the road-side unit is based on a wired or wireless channel. It may co-exist with the road-side unit in a physical cell.

#### 3.1.3. Road-Side Unit

As a wave device, the road-side unit is often equipped along two sides of the road or in some special places, such as road junctions or neighboring parking lots. On the one hand, it can be used to expand the communication scope of VANETs by redistributing messages to other on-board units and transmitting messages to other road-side units; on the other hand, it can be used for some secure applications—for example, early warning of traffic accidents or low bridges. Furthermore, it also can be used to connect to the Internet.

### 3.2. Communication Domains

**Figure 4:**
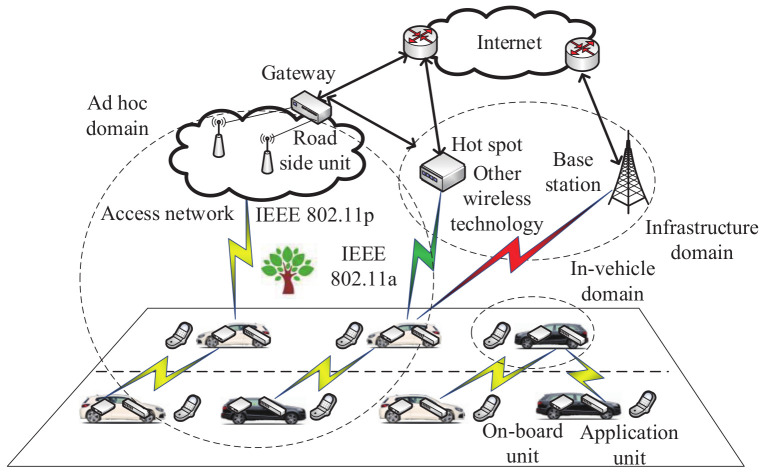
Communication domains in vehicular ad hoc networks.

As shown in Figure 4, there are three kinds of communication domains, namely the in-vehicle domain, ad hoc domain and infrastructural domain. They are described as follows.

#### 3.2.1. In-Vehicle Domain

The on-board unit and application unit are in this domain. The communication can be wired or wireless. The wireless communication is based on the technology of a wireless universal serial bus or ultra-wideband. In addition, the on-board unit offers a communication chain to the application unit.

#### 3.2.2. Ad Hoc Domain

In the ad hoc domain are multiple vehicles that are allocated with on-board units and the road-side unit. In addition, there are two kinds of communications [71] in this domain; they are described as follows.

Inter-vehicle communication [71,72,73,74] is helpful to improve the security of public traffic, increase driving efficiency and enhance the view of on-board devices. Thus, it has attracted attention from academical researchers and companies, especially in the United States, European Union and Japan. In inter-vehicle communication, vehicles are connected by on-board units. If there is an available wireless link between a vehicle and another vehicle, they can communicate with each other. Vehicle-to-vehicle communication is only one-hop. Otherwise, the communication between two vehicles needs a special routing protocol, and then vehicle-to-vehicle communication can be multi-hop.

For the sake of the expansion of communication distance, vehicles are connected with the road-side unit by transmitting, accepting and relaying messages. In addition, with the help of the road-side unit, the vehicle can implement some special applications.

#### 3.2.3. Infrastructural Domain

The road-side unit can be connected with the infrastructural domain. Then, the on-board unit can access the infrastructural domain. In addition, the on-board unit can be connected with some hosts by cellular radio networks, including universal mobile telecommunications system, worldwide interoperability for microwave access (WiMAX), high speed downlink packet access and general packet radio service.

### 3.3. Wireless Access Technologies

There are currently numerous usable wireless access technologies that can be used for vehicle-to-vehicle and vehicle-to-infrastructure communications. The target of these communication methods is to guarantee secure traffic and efficient transportation. In VANETs, common wireless access technologies include dedicated short range communications, fourth generation (4G)/fifth generation (5G) cellular networks, WLAN and worldwide interoperability for microwave access (WiMAX). These communication technologies are described as follows.

#### 3.3.1. Dedicated Short Range Communications

Most vehicular communications are based on dedicated short range communications (DSRC) [75,76,77]. These are particularly designed for achieving low latency and high reliability in vehicle-to-vehicle (V2V) and vehicle-to-infrastructure (V2I) communications [78,79]. The DSRC protocol stack mainly consists of the IEEE 802.11p family [70,80,81,82], which is called wireless access in vehicular environments (WAVE), and the IEEE 1609 family. Detailed protocols can be found in [83]. In DSRC, there are some characteristics that are different from those of other networks [84,85,86]. These characteristics include a real-time guarantee, high mobility, rapidly changing topology, multi-hop communication, limited bandwidth, etc.

#### 3.3.2. 4G/5G Cellular Networks

Fourth-generation cellular networks [87,88] can provide services for mobile ultra-broadband internet access. Users have access to various networks without changing from one network to another network manually. In order to realize fast transmission in some specified regions, some available technologies include long-term evolution-advanced mobile communication systems and microcell base stations. As supported by Internet protocol version 6, the data transmission speed can be up to 1 gigabit per second for communication with low mobility. In addition, the transmission cost per bit of multimedia service is low. Due to users’ various requirements, the service should be quite differentiated, meaning that users can enjoy the benefits of different kinds of services simultaneously.

In 2018, the organization of the third-generation partnership project defined 5G cellular networks [89,90,91], which are extensions of 4G cellular networks. In 5G cellular networks, a large amount of multi-input multi-output antennas are integrated into base stations. For wireless transmission, the technique of millimeter wave communication is used to offer a bandwidth with a frequency of hundreds of megahertz (MHz). Fifth-generation cellular networks have several characteristics, which are described as follows. The data transmission rate of 5G cellular networks can be up to 10 gigabits per second, which is 100 times faster than that of 4G cellular networks. The latency of 5G cellular networks is less than 1 millisecond, which is less than that of 4G cellular networks. In 5G cellular networks, multi-connectivity [92] enables each user to maintain multiple possible signal paths to different cells. Therefore, it is helpful for coping with link failures and the throughput degradation of cell-edge users. Fifth-generation cellular networks also support network slicing [93], which can be used to provide on-demand tailored services for different application scenarios in the same physical network. Furthermore, 5G cellular networks support service differentiation [94]; for example, 5G cellular networks provide three generic services [95], namely Enhanced Mobile Broadband (eMBB), Ultra Reliable Low Latency Communications (URLLC) and Massive Machine Type Communications (mMTC). eMBB supports stable connections with very high peak data rates, URLLC has a low packet delay and high reliability and mMTC supports a large number of devices from the Internet of Things. Thus, 4G/5G cellular networks are helpful for the construction of VANETs.

#### 3.3.3. WLAN

WLAN [96,97,98] is a wireless communication technology which can provide a link to the wider Internet by access points with high flexibility. In WLAN, the coverage of each access point is about 100 m, which is enough to cover the area of a medical institution. The data transmission rate is tens of million bits per second. With the appearance of IEEE 802.11 standards, WLAN is becoming increasingly popular for the research of VANETs.

#### 3.3.4. WiMAX

WiMAX [99,100,101] is a wireless broadband communication technology which is designed based on the IEEE 802.16 standard [102]. In WiMAX, the coverage range of wireless broadband access is at most 50 kilometres (km) for fixed stations. For mobile stations, the coverage range of wireless broadband access is between 5 km and 15 km. The frequency of WiMAX is between 2 gigahertz (GHz) and 66 GHz. It can offer wireless broadband access for two fixed mobile networks.

#### 3.3.5. Satellite Communication

Satellite communication [103,104,105] can transmit and magnify telecommunication signals. This communication technique usually consists of a satellite, ground stations, smart phones, laptops, etc. It can establish communication channels between signal senders and receivers, which are distributed at different places around the world. In satellite communication, the data rate of the downlink is at most 1000 gigabits per second, and the maximum data rate of the uplink is 1000 mbps. Its coverage range is between 100 km and 6000 km. Satellite communication is helpful for the development of VANETs.

### 3.4. Cyber-Security Issues

In the environment of VANETs, cyber-security issues can be mainly classified into in-vehicle domain attacks, ad hoc domain attacks, infrastructure domain attacks and insecure data analysis. These attacks and their corresponding solutions are described as follows.

In-vehicle domain attacks mainly include GPS spoofing attacks [106] and close-proximity vulnerability attacks. In GPS spoofing attacks, the adversary broadcasts fake GPS signals whose strength is stronger than that of the true GPS signals. Therefore, GPS receivers will ignore the true signal. These attacks can be detected by various techniques, such as statistical properties [107], a Kalman filter [108] and phase delay measurements [109]. Based on the vulnerabilities in the Bluetooth-enabled system [110] and keyless entry code, close proximity vulnerability attacks can be used to interfere with the vehicle’s engine control units or gain access to a vehicle. In order to defend against these attacks, commercial products often use open-source tools; for example, Bluesniff [111].

Ad hoc domain attacks consist of denial of service attacks [112], impersonation attacks [113], replay attacks [114], routing attacks [115], eavesdropping attacks [116], etc. In DoS attacks, the adversary blocks the whole communication channel with interference signals. Then, authentic users cannot access network services. To resist these attacks, some detection strategies [117,118,119] are performed by the road-side unit or on-board unit. Impersonation attacks are implemented by using another identity or a fake identity. To defend against these attacks, the key is to determine the actual position of the vehicle from which the message originated. Replay attacks may confuse the authorities, mislead the entire traffic or even decrease transportation safety. In order to thwart these attacks, common methods include the session key [120] and proxy signature [121].

In routing attacks, an adversary can disturb the normal routing process or drop passing packets. In order to detect these attacks, there are several available defense strategies, including the watchdog technique [122], trust-based method [123] and self studying [124]. Eavesdropping attacks are implemented by listening to the wireless medium. In these attacks, an adversary can collect a vehicle’s private positions and activities silently. In order to prevent these attacks, encryption is a common method; in addition, the key needs to be constantly changed.

In infrastructure domain attacks, the adversary may take control of a traffic light and signal. Then, a false signal phase will be sent to the vehicle, which will threaten its security. Besides this, VANET applications require interactions with the Cloud server in the infrastructure domain. This will introduce cyber-security concerns, including data protection, cyber attacks and compliance with privacy regulations. In order to detect and mitigate against an infrastructure domain attack, Mashrur et al. [125] utilized a Cloud-based method. In order to provide sufficient levels of protection for the infrastructure of VANETs, software-defined security [126] is used by abstracting security mechanisms from the hardware layer to a software layer.

In VANETs, for the efficient implementation of data analysis, vehicle data should be transmitted to the Cloud server, as the Cloud server is responsible for the computation of vehicle data. Because the Cloud server is untrusted, vehicle data may be illegally accessed, forged, tampered with or discarded in the process of transmission and computation. For example, Qayyum et al. [127] analyzed extant adversarial machine learning attacks in VANETs. In order to defend against insecure data analysis, there are some efficient solutions, which are described as follows. Zhu et al. [128] combined secure multi-party computation and dynamic k-anonymity. The anonymity parameter k is dynamically decided by the vehicle density, anticipated anonymous region and network topology. Riazi et al. [129] presented a novel hybrid framework for secure function evaluation, which enabled two parties to jointly compute a function without disclosing their private inputs.

Besides this, for the security of VANETs, there are several cyber-security and safety standards, including International Organization for Standardization (ISO) 26262, Society of Automotive Engineers (SAE) J3061 and the British Standards Institute’s cyber security standard. Details of these standards can be found in [130,131,132,133,134].

## 4. Homomorphic Encryption-Based Secure Computation in Vehicular Ad Hoc Networks

Based on homomorphic encryption, we first present the current state of secure basic operations, data aggregation, data query and other data computation methods in VANETs. Then, we present several challenges and open problems for future research.

### 4.1. Basic Operations

In order to implement homomorphic encryption-based secure computation for VANETs, it is necessary to study basic operations including comparison, division, inner product, set operations, etc. There are some available secure basic operations, which are described as follows.

Dou et al. [135] first proposed an encoding technique which can convert a private set to a private vector. Then, the secure subset problem can be transformed to the computations of private vectors. Based on this encoding technique and the Paillier scheme, the authors designed a novel and efficient private subset computation protocol, which is secure in a semi-honest model or malicious model. Dou et al. [136] first designed a new encoding technique which can convert user data to a special vector. Based on this encoding technique and ElGamal scheme, Dou et al. [136] proposed a secure minimum protocol for the computation of the minimum of several numbers privately. This protocol is secure in the semi-honest model. In addition, this protocol can be used for the secure computation of the maximum and union of sets. Liu et al. [137] designed a vector encoding method which can convert a number to a vector. Then, the comparison problem can be transformed into the computation of the vector. Based on this encoding method and GM algorithm [22], the authors proposed a privacy-preserving comparison protocol for integers. This protocol requires 6L+4 modular multiplications, where *L* is the length of the vector. The communication cost of this protocol is at most two rounds. In order to compare rational numbers securely, the authors designed a protocol which utilized a geometric method. This protocol is information-theoretically secure.

Liu et al. [138] used the vectorization method to transform the greater-than problem into the computation of the vector. Then, based on the Paillier algorithm, the authors designed a secure protocol that could solve the greater-than problem in one execution. This protocol reqzures 2(s+2)lgq modular multiplications, where *q* is the modulus in the Paillier scheme and *s* is the dimension of vector. The communication cost of this protocol is only one round. Inspired by computational geometry, Li et al. [139] first proposed a method which could transform the comparison problem of two rational numbers into the computation of the area of a triangle, which is formed by three private points. Based on this method and the Paillier algorithm, the authors proposed a secure comparison protocol for rational numbers. This protocol has low computational complexity.

In 2018, Gong et al. [140] proposed an efficient method that could compare two integers *a* and *b* by computing the formula $\left(ka+k_{1}\right)/\left(kb+k_{1}\right)$, where *k*, $k_{1}$ are additional two integers. Then, based on this method and the Paillier algorithm, the authors designed two efficient protocols for secure comparison on integers and fractions, respectively. In order to reduce computational complexity, the proposed protocols outsource the time-consuming exponent calculation to the Cloud server in the phase of data pretreatment. Based on the Paillier algorithm with threshold decryption, Liu et al. [141] designed secure outsourced calculation toolkits for integers and rational numbers. These toolkits consist of basic operations, which include multiplication, division, comparison, sorting, equivalence testing and greatest common divisor. The authors also designed a secure reducing fraction protocol that could securely reduce the greatest common divisor of the numerator and denominator. Security analysis shows that that these protocols are secure in the semi-honest model. Huang et al. [142] first proposed a new, secure, scalar product method, which include four phases: input, calculation, output and proof of correctness. Then, based on this method and the Paillier algorithm, the authors constructed a secure Euclidean distance protocol which satisfied the network delay, computation and communication complexity. This protocol supports rational numbers; in addition, this protocol is secure in the semi-honest model.

For homomorphic encryption-based secure computation in VANETs, there are several available schemes, including data aggregation, data query and other data computation methods. They are compared in Table 1.

### 4.2. Data Aggregation

In VANETs, data aggregation [163,164,165,166,167] is used to decrease the amount of vehicle data. This is helpful for increasing the efficiency of data transmission. Thus, based on the Paillier algorithm or other homomorphic encryption algorithms, data aggregation is applied in the following schemes.

#### 4.2.1. Paillier Algorithm

In this section, data aggregation based on the Paillier algorithm is implemented in several schemes, which are described as follows.

**Figure 5:**
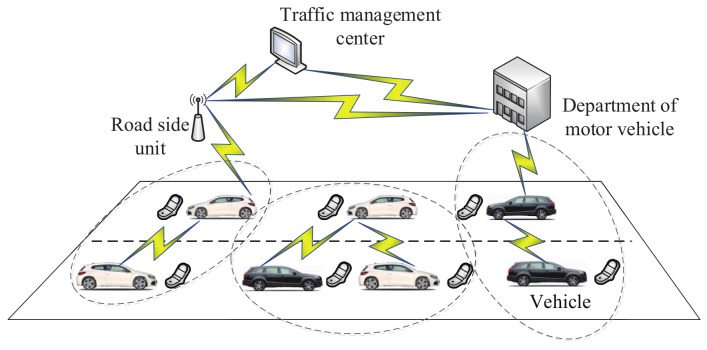
The network model in [143].

In order to protect the privacy of vehicles, Rabieh et al. [143] proposed a secure routing report mechanism in VANETs. As shown in the network model (Figure 5) of this mechanism, there are four entities: vehicles, road-side units, the traffic management center and department of motor vehicles. Road-side units are connected with the traffic management center by WiMAX, 4G or another fast communication technology. Encrypted routing data, which are based on segments, are offered to road-side units. In addition, based on the Paillier algorithm, vehicle data are aggregated by road-side units. Then, aggregated data are transmitted to the traffic management center, which will acquire the amount of vehicles in every segment without leaking vehicles’ private information. It has been demonstrated that this mechanism has acceptable costs in terms of communication and computation.

In 2016, based on the Paillier algorithm, Rabieh et al. [144] proposed a secure route-sharing method for VANETs. In this method, there is a vehicle called the leader, and other vehicles are regarded as subleaders. The leader needs to send a request to create a platoon of vehicles. The leader and subleaders create public keys and corresponding secret keys. Then, every vehicle adds a random number to the route secretly. The processed route is encrypted by the public key. The ciphertext is returned to the leader. Next, the same random number is deducted from the route by all the vehicles in the platoon. The encrypted processed route is sent to the subleader. The leader and subleader will aggregate messages and will decrypt the aggregated message. Last but not least, decryptions are exchanged to obtain the aggregated routes. In order to simulate the proposed method, the IEEE 802.11p standard was chosen as the communication technology among vehicles. The experimental results showed that this method has acceptable costs in terms of computation and communication.

**Figure 6:**
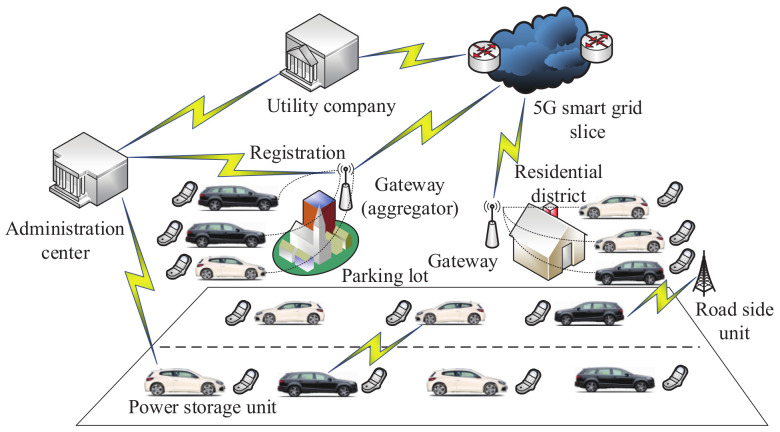
The architecture of the secure communication and power injection scheme.

In 2018, based on the Paillier algorithm, Zhang et al. [145] constructed a secure communication and power injection scheme which is suitable for autonomous vehicle networks and 5G smart grid slices. As shown in Figure 6, the architecture of this scheme consists of the utility company, the administration center, the road-side unit, the power storage unit and various communities, which include the parking lot and the residential district. In a community, the communication between power storage units relies on the gateway. Each gateway communicates with the utility company by way of the 5G smart grid slice. In addition, the utility company can only obtain the overall quantity of power injected by power storage units. Separate power bids cannot be seen. Based on the hash-then-homomorphic technology and Paillier algorithm, power injection bids, which are related to the time slots, are aggregated and blinded by each electrical vehicle.

**Figure 7:**
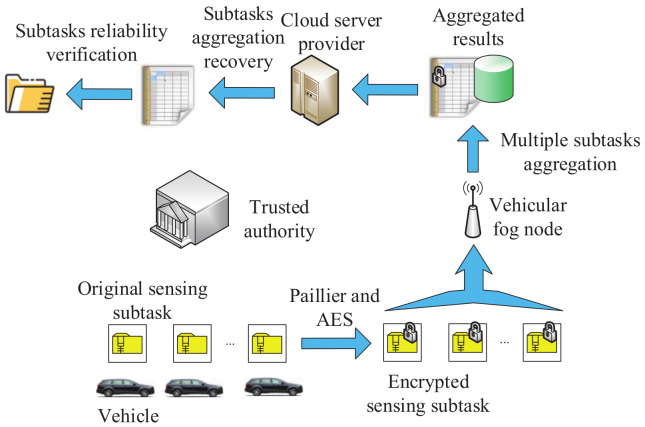
The architecture of the secure task recomposition method.

In 2018, based on the Paillier algorithm, Wang et al. [146] proposed a secure task recomposition method for crowdsensing in a vehicular fog computing system. As shown in the architecture (Figure 7) of this method, there are vehicles with limited capability of communication and computation, the vehicular fog node, the Cloud service provider and the trusted authority. Dedicated short-range communication and 5G mobile communication systems can be used for the communication among these entities. Hybrid subtasks are aggregated into a ciphertext. Every sensed subtask is encrypted by the Paillier algorithm and advanced encryption standard (AES). Then, it is transmitted to a surrounding vehicular fog node. Next, encrypted subtasks are converted to new ciphertexts, which are only encrypted by the Paillier algorithm. The vehicular fog node will aggregate all the collected subtasks. The aggregated result is sent to the Cloud service provider. Based on the received aggregated results, the Cloud service provider recovers the aggregation of every subtask and tests the reliability. The experimental results show that the proposed method has acceptable costs in terms of computation and communication.

**Figure 8:**
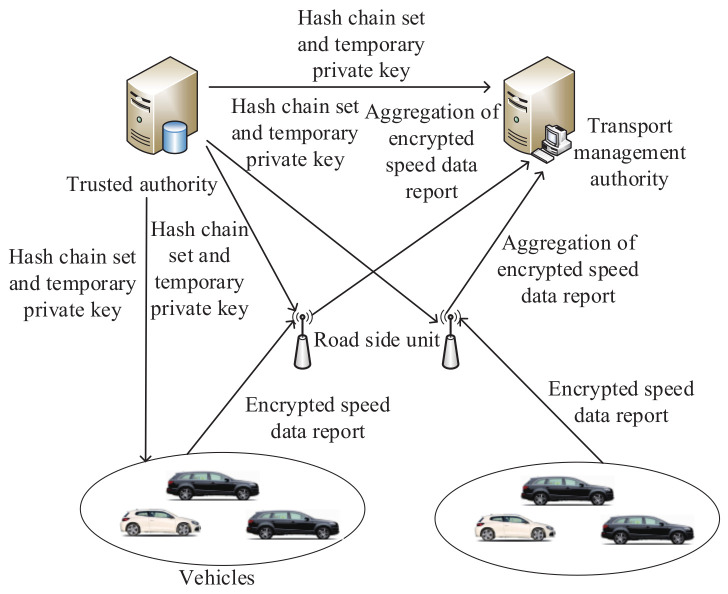
The architecture of the secure and autonomous analysis mechanism for traffic movement.

In 2018, in order to protect the security of the intelligent transportation system, Ogundoyin [147] designed a secure and autonomous analysis mechanism for traffic movement. In this mechanism, traffic data, which include the average speed of vehicles, can be acquired without leaking user privacy. Based on the improved Paillier algorithm and Chinese remainder theorem, various roads’ data are aggregated. Then, bandwidth and authentication time will be saved. As shown in Figure 8, the proposed mechanism includes four entities: the trusted authority, the transport management authority, road-side units and vehicles. Furthermore, the communication between the trusted authority and vehicles relies on a secure link; for example, a secure socket layer. Road-side units can communicate with vehicles by the IEEE 802.11p standard. In the node registration, the trusted authority will output the hash chain set and temporary private key. Then, they will be sent to other entities. The road-side unit executes the aggregation of the encrypted speed data report originating from vehicles. Next, the transport management authority can analyze these aggregated data.

**Figure 9:**
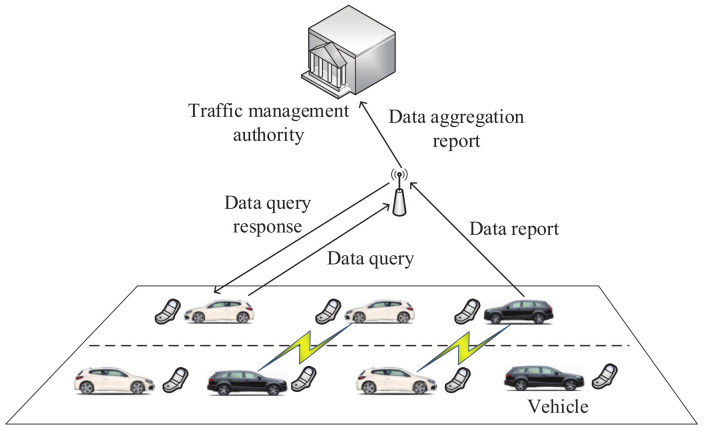
The architecture of the secure data sharing scheme.

In 2019, Kong et al. [148] first proposed a secure data sharing scheme for the Internet of Vehicles; this method can significantly reduce the burdens on system resources. As shown in the architecture of this scheme (Figure 9), the method includes vehicles, road-side units and the traffic management authority. Every vehicle builds a composite data report. Next, the data report is sent to road-side units. Based on a modified Paillier algorithm, road-side units implement the secure data aggregation. Then, the aggregated result is transmitted to the traffic management authority. If road-side units receive a data query from a vehicle, the aggregated result will be shared with the vehicle. In the process of data querying, the wireless communication between road-side units and various vehicles relies on the IEEE 802.11p standard.

#### 4.2.2. Other Algorithms

In this section, based on other homomorphic encryption algorithms, data aggregation is implemented in several schemes, which are described as follows.

**Figure 10:**
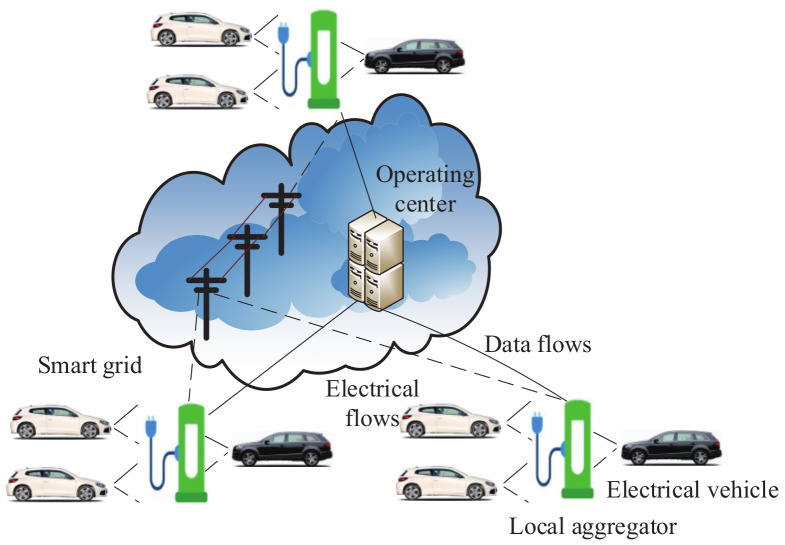
The architecture of vehicle-to-grid networks in a smart grid.

**Figure 11:**
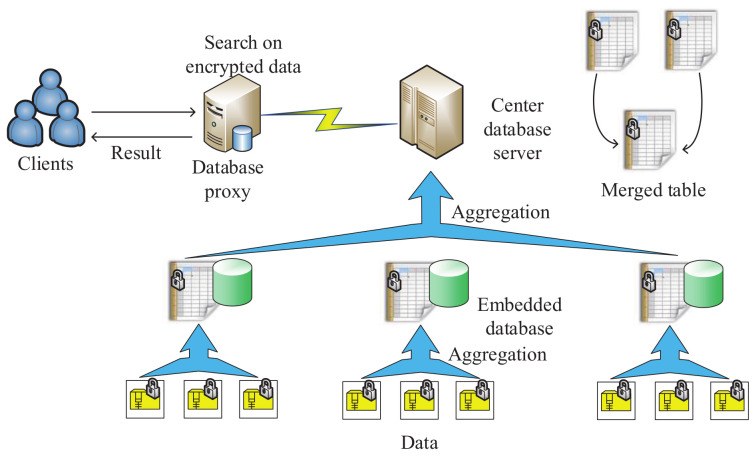
The architecture of the secure data management framework.

In 2016, for the security of data aggregation and data publication in smart grid vehicle-to-grid networks (Figure 10), Han et al. [149] proposed a secure data management framework based on Ozdemir’s homomorphic encryption scheme [150]. As shown in the architecture of this framework (Figure 11), the method includes clients, a database proxy, a central database server and an embedded database. The embedded database is helpful for storing the encrypted data of electrical vehicles. Then, local aggregation is implemented on these encrypted data. Next, based on these local aggregation results, the central database server calculates the final aggregation result. If the client sends a query to the central database server, the database proxy will hide private information in the query. The central database server executes the processed query, and the encrypted query result is returned to the client. Furthermore, the security of this framework was analyzed under some classical cyber attacks, including the replay attack, interface attack, known plaintext attack, etc. The experimental results showed that the proposed framework is efficient.

In 2019, Prema [151] first proposed a modified FHE scheme. In this scheme, re-encryption is used to reduce the computational cost. Compared with the Paillier algorithm, this scheme requires less communication overhead to transmit messages. Then, the author used this scheme to construct an efficient and secure aggregation protocol which is helpful for aggregating data in VANETs. In this protocol, mutual authentication is used for the utilization of the data service and access server in VANETs. Furthermore, a self-generated pseudonym is created in the process of authentication. Next, the road-side unit can broadcast a self-generated pseudonym; this protocol can avoid leaking distance estimation by other nodes. In addition, it can avoid being attacked by malicious nodes.

### 4.3. Data Query

According to the special requirements of users, data query [168,169,170,171,172] is used to search appropriate vehicle data from a database table in VANETs. Thus, data query has been applied in several schemes, which are described as follows.

**Figure 12:**
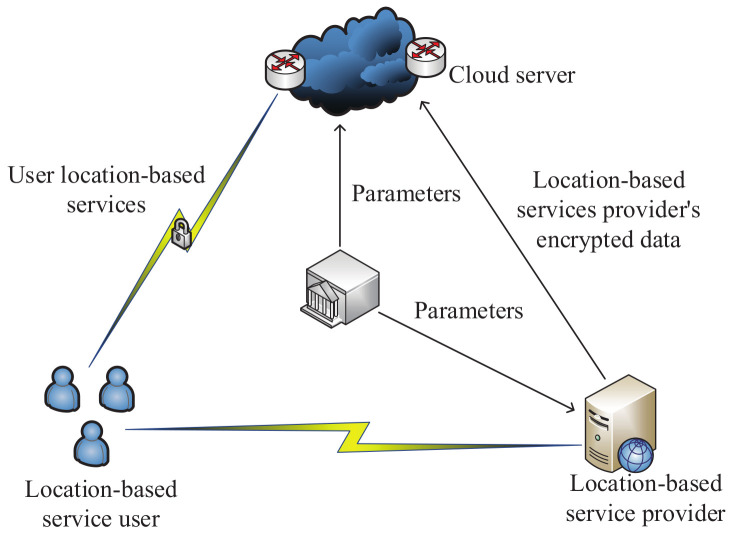
The architecture of the secure polygon spatial query scheme.

In 2016, with the aim of searching through data of location-based services securely, Zhu et al. [152] proposed a secure polygon spatial query scheme, which is based on an improved 2-DNF algorithm [25]. As shown in the architecture of this scheme (Figure 12), the participating entities include the authority, location-based services provider, Cloud server and location-based services user. The authority first initializes the entire scheme by generating parameters that are transmitted to the Cloud server and location-based service provider individually. Next, the location-based services provider’s data are encrypted. In addition, ciphertexts are outsourced to the Cloud server. Then, a location-based services user can search any polygonal area to obtain accurate results. In order to evaluate the performance of the proposed scheme, it was implemented on a mobile phone and three workstations. They could communicate with each other by IEEE 802.11g WLAN technology. The experimental results demonstrated the efficiency of the proposed scheme.

**Figure 13:**
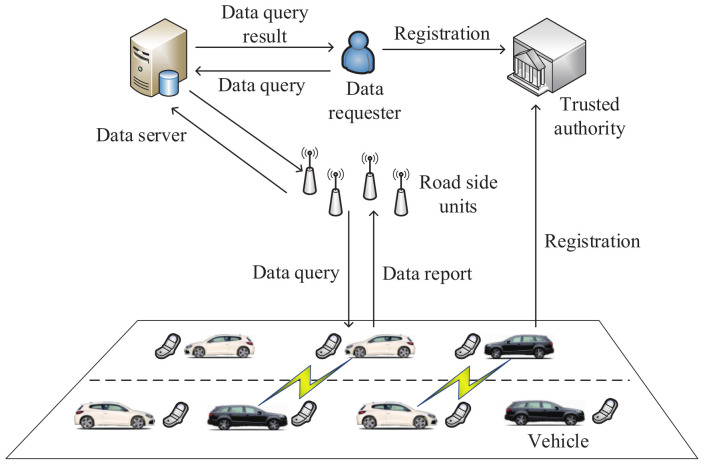
The architecture of the secure range query method.

In 2017, Kong et al. [153] designed a secure range query method from distributed on-board storage in VANETs. In this method, the secure computation of scalar products is based on the Paillier algorithm. In addition, every multi-dimensional scale is structured into one dimension. As shown in Figure 13, the method includes vehicles, the data requester, the data server, the trusted authority and road-side units in the architecture of this query method. The wireless communication between vehicles and road-side units relies on the IEEE 802.11p standard. If the data server receives a data query, it will be forwarded to all vehicles by road-side units. When the data server receives a data report, it will execute the process of data filtering. Then, the data query result is returned to the data requester. Simulation results show that the proposed method can significantly reduce the costs of communication and computation.

**Figure 14:**
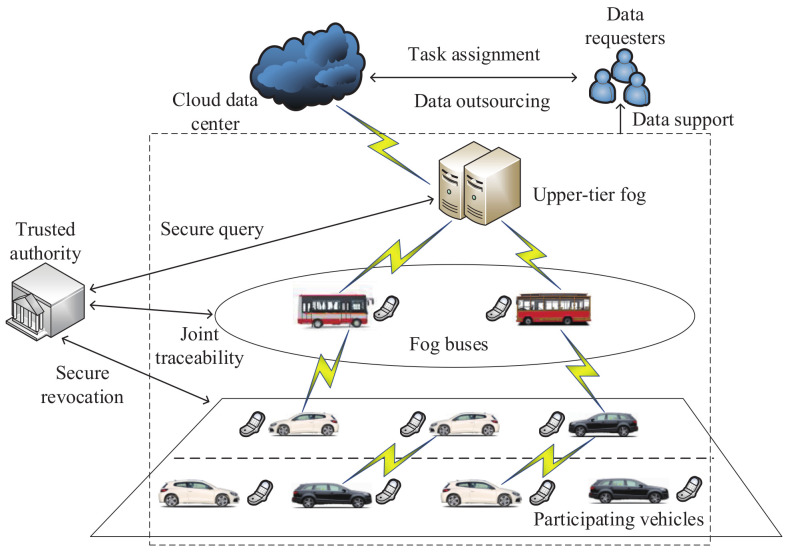
The architecture of the synthetic vehicle crowdsensing scheme.

In 2019, based on the fog computing technology, Sun et al. [154] designed a new synthetic vehicle crowdsensing scheme for the security of data collection. As shown in the architecture of this scheme (Figure 14), the method includes data requesters, participating vehicles, fog buses, upper-tier fog and the Cloud data center. Task assignment and data outsourcing are carried out between data requesters and the Cloud data center. The implementation of the secure query, joint traceability and secure revocation requires a trusted authority. A data support service can be provided by participating vehicles, fog buses and upper-tier fog. Participating vehicles have a wireless communication module that relies on device-to-device communication technologies such as WiFi and dedicated short-range communication. For the security of participating vehicles and data requesters, the authors used a two-tier fog architecture with a secure data aggregation scheme, which was based on the Paillier algorithm and 2-DNF algorithm [25]. Then, this aggregation scheme was simulated by IEEE 802.11p communication technology. Evaluation metrics included throughput, the participation ratio and the successful participation ratio. In addition, the simulation results showed that the proposed aggregation scheme is suitable for urban districts.

In 2018, based on the Paillier algorithm, He et al. [155] constructed a secure three-step ride-matching scheme in ride-sharing services. In this scheme, the authors first proposed a secure selection method based on spatial regions. Next, the ride-sharing server chooses possible ride-sharing partners with the aim of saving travel time and improving the practicability of time arrangement. Then, in order to maximize the system-wide travel time savings, the ride-sharing server selects suitable ride-sharing partners. In the final experiment, the proposed scheme was implemented on several Nexus 5 mobile phones, the operating system of which was Android 6.0. In addition, the communication technique relied on Bluetooth 4.0 [173,174], the transmission rate of which is more than 900 kilobyte per second. Thus, the communication overhead had less impact on the execution time. The simulation results showed that the proposed scheme is efficient and practical without leaking private data.

**Figure 15:**
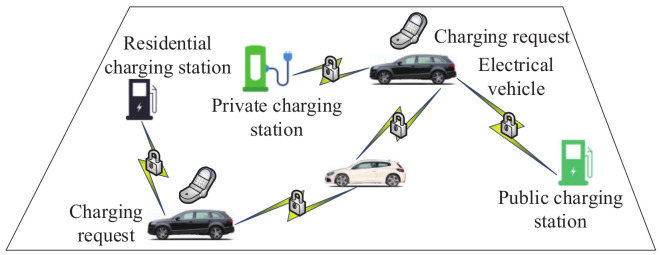
The architecture of the matching system.

In order to protect sensitive data of electrical vehicle drivers, based on the Paillier scheme, Yucel first et al. [156] proposed a secure bichromatic mutual nearest neighbor computation method using peer-to-peer communication technology; for example, dedicated short-range communication technology. Then, the authors designed an efficient and secure distributed online matching system by the proposed computation method. As shown in Figure 15, the method includes electrical vehicles and charge suppliers, which include a public charging station, private charging station, residential charging station and vehicle-to-vehicle charge supplier. The electrical vehicle first starts a local query to check whether there are surrounding charge suppliers. If there are available charge suppliers nearby, the electrical vehicle will send a charging request. In order to match the charging request in a distributed way, these charge suppliers will reply to the electrical vehicle in a reasonable time.

In the process of sharing autonomous vehicles, private data of vehicles may be leaked [175]. In order to avoid this issue, Hadian et al. [157] proposed a secure time-sharing method based on the Paillier algorithm. In this method, a secure matching task can be executed without leaking the vehicle’s location information and route data. In order to rent a vehicle, the requesters upload their encrypted requests to the server, where the encrypted requests include the travel time, pickup location and drop-off location. According to the requested locations and time, the server calculates the corresponding cost values. Next, they are transmitted to vehicle owners. Based on cost values, the vehicle owner chooses the requester with the minimum cost value. Finally, the server creates a direct communication channel between the vehicle owner and the chosen requester.

In 2018, for the security of vehicle-to-everything communications, Ulybyshev et al. [158] designed a secure data exchange mechanism which offers access control, which is based on roles and attributes. This method can detect and prevent data leakage caused by insiders. Then, based on partial homomorphic encryption, the authors proposed an encrypted search method which can query the ciphertexts of vehicle records, which are usually stored on an untrustworthy Cloud server. In addition, this method supports the subset of structured query language queries on the ciphertexts. This kind of query can be utilized to filter-out related vehicle records in the early stage of the data request. Next, the data request is transmitted to related vehicle records. In the final experiment, the communication between two vehicular nodes is based on the transmission control protocol/Internet protocol. The time interval of the data transaction implemented between two vehicular nodes is 152 milliseconds.

### 4.4. Other Data Computation

In VANETs, other types of data computation include verification [176,177,178], tendering mechanism, etc. They have been studied in several schemes, which are described as follows.

**Figure 16:**
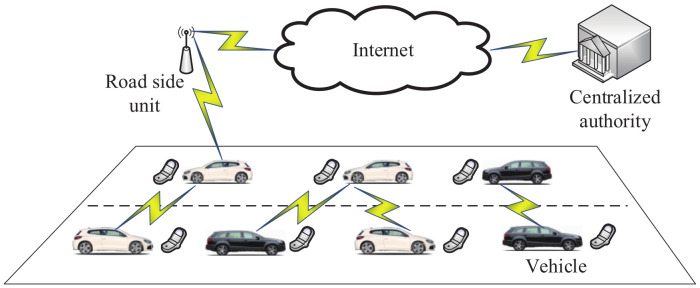
The network model in [159].

In order to protect drivers’ privacy, Rabieh et al. [159] proposed a secure chatting mechanism. In this mechanism, attribute-based encryption is used to verify common interests anonymously. In addition, partial homomorphic encryption technology is used to verify the degree of interest anonymously. As shown in Figure 16, the network model of this mechanism includes three entities: vehicles, the centralized authority and road-side units. The centralized authority is used to generate secret keys, update the interests of drivers and revoke the keys of interests. Road-side units are connected with the centralized authority by WiMAX, 4G or other communication technology. Furthermore, the authors designed a secure search scheme that is helpful for checking vehicles’ common interests. This scheme has low costs in terms of communication and computation.

**Figure 17:**
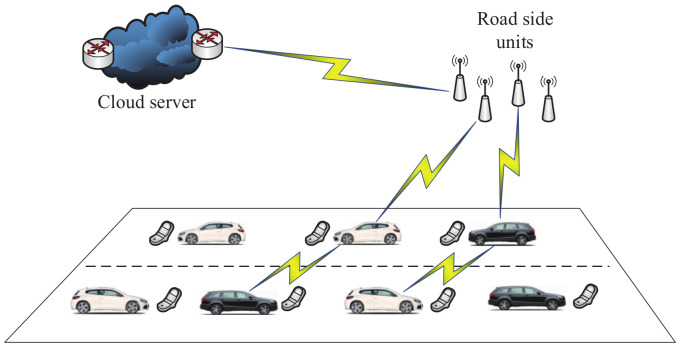
The architecture of the secure incentive mechanism.

In 2017, based on fully homomorphic encryption, Alamer et al. [160] designed a secure tendering mechanism in the vehicular Cloud. As shown in Figure 17, a tendering framework is used to model the interaction between the Cloud server and vehicles. In addition, the Cloud server selects vehicles to collaborate in the implementation of announced tasks. If there is a task, the Cloud server will advertise it to a road-side unit. Based on the task, the road-side unit broadcasts the packet of resource procurement to the vehicles under its scope. If some vehicles want to participate in the task, their tenders will be used for reply. Based on the tenders, the Cloud server and road-side unit choose vehicles and their rewards. Then, the chosen vehicles execute the task and offer corresponding resources to the Cloud server. Each vehicle is rewarded for its tender. Finally, the properties of this mechanism are evaluated by extensive simulations.

**Figure 18:**
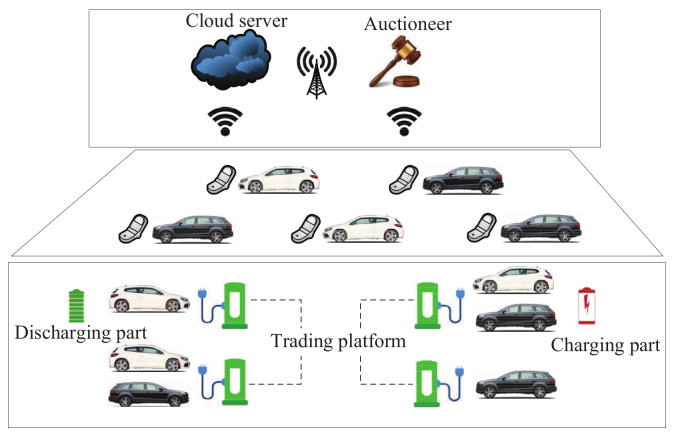
The architecture of the secure double auction scheme.

Vehicle-to-grid technology, which is based on the communication between electrical vehicles and the smart grid, can provide demand response services by delivering electrical energy to the smart grid. In order to protect user privacy, based on Boneh’s algorithm [25], Li et al. [161] designed a secure double auction scheme for the demand response of electrical vehicles in microgrid outages. As shown in Figure 18, the architecture of this scheme includes the Cloud server and the auctioneer. The auctioneer can be used to solve the problem of maximizing social welfare. Energy transactions can be negotiated between purchasers and sellers. The Cloud server is regarded as a middleman between bidders and the auctioneer. In this scheme, electrical vehicles that have redundant electrical energy are regarded as sellers. They can release their electrical energy by vehicle-to-grid technology. If electrical vehicles do not have sufficient electrical energy, they will act as purchasers. This scheme can be executed whenever there are both purchasers and sellers. In addition, it has acceptable costs in terms ofof computation and communication.

**Figure 19:**
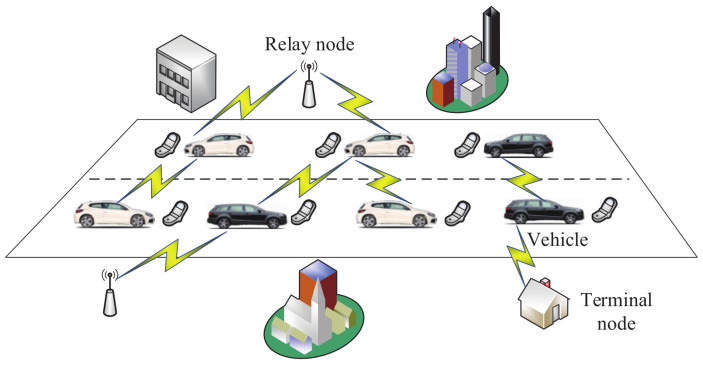
The model of vehicular delay-tolerant networks.

In 2018, Magaia et al. [162] designed a secure opportunistic routing protocol. In this protocol, vehicular delay-tolerant networks (Figure 19) [179] are modeled as time-varying neighborhood graphs, where edges are regarded as the relationship of two neighboring vehicles. This protocol consists of the generation and anonymization of the neighborhood graph, routing algorithm, etc. In the routing algorithm, the exchange of messages is based on the Paillier algorithm and the process of routing decisions. Finally, this protocol is simulated by the opportunistic network environment simulator in various simulation cases, which include synthetic mobility models and real mobility tracing. In synthetic mobility models, all the nodes are supposed to use interfaces of Bluetooth and IEEE 802.11a WiFi; however, the nodes use the interface of IEEE 802.11p WiFi in real mobility tracing. The simulation results showed that the cryptographical cost of this protocol is low in most cases.

### 4.5. Challenges and Future Research Directions

The idea of homomorphic encryption-based secure computation has been widely accepted. Several secure computation methods based on homomorphic encryption for VANETs have been developed; these methods are helpful for the research of the secure computation of vehicle data. In this field, the research has mainly focused on the achievement of complicated operations by consuming fewer computing resources. Moreover, secure computation should support multiple types of datasets. There are still some problems in the research into the secure computation of vehicle data; these problems are described as follows.

Most of the above schemes adopted general homomorphic encryption schemes. In addition, they seldom considered the precision of vehicle data and the depth of multiplicative homomorphic methods. Thus, corresponding parameters of schemes cannot be set up. This is not helpful for the realization of an efficient secure computation method for vehicle data.

With the aim of constructing a secure computation method for vehicle data, most protocols have been built based on partial homomorphic encryption or FHE. Partial homomorphic encryption only supports homomorphic addition or homomorphic multiplication; it requires additional rounds of interaction to implement homomorphic addition or homomorphic multiplication. Although FHE supports an infinite number of homomorphic addition and homomorphic multiplication operations, the running time of homomorphic multiplication is excessively long. Thus, it is difficult to guarantee real-time communication in VANETs. Furthermore, due to the large sizes of public keys and ciphertext, their transmission may occupy the limited bandwidth in VANETs.

In the secure computation of vehicle data, data aggregation and data searching are commonly used methods. In order to implement these methods, vehicle data should first be encoded. The current usual encoding methods only support integers and floats; thus, attention should be paid to new encoding methods for other complicated data types. Homomorphic encryption only supports homomorphic addition and homomorphic multiplication; thus, the efficient realization of these methods by homomorphic addition and homomorphic multiplication is a challenge. In these algorithms, some complicated operations such as exponentiation and logarithms can be expressed as addition and multiplication by the technique of the Fourier series. Then, they can be implemented by homomorphic operations. However, the efficiency of homomorphic operations cannot be guaranteed if the precision of messages is high.

In the secure computation of vehicle data, multi-user data are encrypted into ciphertexts, which are usually under the same secret key. However, the security of multi-user data may be affected. With the help of extra rounds of interaction, partial homomorphic encryption can support operations on the ciphertexts with different secret keys. We can also use the technique of secure multiparty computation [180] to solve this issue. Multi-key FHE and threshold FHE can be used to design a secure multiparty computation protocol. Although multi-key FHE supports operations on the ciphertexts with different secret keys, unfortunately, its efficiency of homomorphic operations and ciphertext conversion decreases with the increasing depth of homomorphic multiplication and the number of parties. The efficiency of threshold FHE is more efficient than that of multi-key FHE. But threshold FHE requires additional rounds of interaction.

## 5. Conclusions

Homomorphic encryption supports computations on the ciphertext without decryption. In view of the advantage of homomorphic encryption, it is used to construct the secure computation method. Thus, homomorphic encryption-based secure computation is studied in VANETs. In this article, we have presented a survey of secure computation based on homomorphic encryption in VANETs. To begin with, we described the related definitions and the current state of partial homomorphic encryption, fully and somewhat homomorphic encryption. Then, we introduced the framework, communication domains, wireless access technologies and cyber-security issues of VANETs. Finally, we presented the state of the art of secure basic operations, data aggregation, data query and other data computation methods in VANETs. In addition, several challenges and open academic problems have been presented.

In summary, research on homomorphic encryption-based secure computation in VANETs is quite broad, and many challenges lay ahead. Nevertheless, it will be advantageous for the community to swiftly address these challenges and move beyond them. This article attempts to briefly explore how homomorphic encryption-based secure computation works and when it should be used to solve problems in VANETs. We also discuss future research directions that may benefit the pursuit of this goal. We hope that our discussion and exploration here may open a new avenue for the development of homomorphic encryption and shed light on secure computation in VANETs.

## Figures and Tables

**Table 1:** The comparison of homomorphic encryption-based secure computation in vehicular ad hoc networks.

Year	Scheme	Secure Computation Technology	Application Scenario	Function	Feature
2015	[143]	Paillier	Routing report mechanism	Aggregate vehicles’ data	Encrypted routing data, which are based on segment, are offered to road-side units
2016	[144]	Paillier	Route sharing method	Aggregate messages	Decryptions are exchanged to obtain the aggregated routes
2018	[145]	Paillier	Communication and power injection scheme	Aggregate power injectiton bids	The utility company can only obtain the overall quantity of power
2018	[146]	Paillier	Task recomposition method	Aggregate collected subtasks, test the reliability	The sensed subtask is first encrypted by Paillier algorithm and AES
2018	[147]	Improved Paillier	Analysis mechanism	Analyze aggregated data	Save bandwidth and the authentication time
2019	[148]	Modified Paillier scheme	Data sharing scheme	Aggregate and share data	Save system resources
2016	[149]	Ozdemir’s homomorphic encryption scheme [150]	Data management framework	Aggregate data	Center database server calculates the final aggregation result
2019	[151]	Modified FHE scheme	Aggregation protocol	Aggregate data	Avoid leaking distance estimation
2016	[152]	Improved 2-DNF algorithm [25]	Polygons spatial query scheme	Search data	The location-based services user can inquire any polygonal area to obtain accurate results
2017	[153]	Paillier	Range query method	Compute scalar product	Every multi-dimensional scalar is structured into one dimension
2019	[154]	Paillier, 2-DNF	Vehicle crowdsensing scheme	Implement query, joint traceability and revocation	Use a two-tier fog architecture
2018	[155]	Paillier	Ride-matching scheme	Select suitable ride-sharing partners	This scheme is three-step
2019	[156]	Paillier	Online matching system	Match the charging request	This scheme is distributed
2017	[157]	Paillier	Time-sharing method	Implement matching task	The vehicle owner chooses the requester, which has the minimum cost value
2018	[158]	Partial homomorphic encryption	Search method	Query the ciphertexts of vehicle records	Support the subset of structured query language queries on the ciphertexts
2015	[159]	Partial homomorphic encryption	Chatting mechanism	Verify common interest and degree of interest, check vehicles’common interests	Centralized authority is used to generate secret keys, update the interests of drivers and revoke keys of interests
2017	[160]	Fully homomorphic encryption	Tendering mechanism	Decide victorious vehicles and their rewards	The cloud server and selected vehicles collaborate to implement announced tasks
2017	[161]	Boneh’s algorithm [25]	Double auction scheme	Solve the problem of maximizing social welfare	This scheme can be executed whenever there exist both purchasers and sellers
2018	[162]	Paillier	Opportunistic routing protocol	Generate and anonymize the neighborhood graph, routing algorithm	Edges are regarded as the relationship of two neighboring vehicles
